# Web-Based STI/HIV Testing Services Available for Access in Australia: Systematic Search and Analysis

**DOI:** 10.2196/45695

**Published:** 2023-09-22

**Authors:** Ethan Trey Cardwell, Teralynn Ludwick, Christopher Fairley, Christopher Bourne, Shanton Chang, Jane S Hocking, Fabian Y S Kong

**Affiliations:** 1 Melbourne School of Population and Global Health University of Melbourne Melbourne Australia; 2 Melbourne Sexual Health Centre Alfred Health Melbourne Australia; 3 Centre for Population Health New South Wales Health Sydney Australia; 4 School of Computing and Information Systems University of Melbourne Melbourne Australia

**Keywords:** STI/HIV testing, STI/HIV, self-testing, sexual health, web-based STI testing, web-based STI/HIV testing

## Abstract

**Background:**

Sexually transmitted infection (STI) rates continue to rise in Australia, and timely access to testing and treatment is crucial to reduce transmission. Web-based services have been viewed as a way to improve timely access to STI/HIV testing and have proliferated in recent years. However, the regulation of these services in Australia is minimal, leading to concerns about their quality. The purpose of this review was to systematically identify web-based STI/HIV testing services available in Australia and assess them on aspects of quality, reliability, and accessibility.

**Objective:**

We aim to systematically identify and assess web-based STI/HIV testing services available in Australia.

**Methods:**

A Google search of Australian web-based services was conducted in March 2022 and repeated in September 2022 using Boolean operators and search terms related to test services (eg, on the internet or home), STIs (eg, chlamydia or gonorrhea), and test type (eg, self-test). The first 10 pages were assessed, and services were categorized as self-testing (ST; test at home), self-sampling (SS; sample at home and return to laboratory), or self-navigated pathology (SNP; specimens collected at pathology center). Website reliability was assessed against the Health on the Net Foundation code of conduct, and service quality was assessed using a scorecard that was developed based on similar reviews, Australian guidelines for in-person services, and UK standards. Additionally, we looked at measures of accessibility including cost, rural access, and time to test results.

**Results:**

Seventeen services were identified (8 ST, 2 SS, and 7 SNP). Only 4 services offered recommended testing for all 4 infections (chlamydia, gonorrhea, syphilis, and HIV) including genital, anorectal, and oropharyngeal sites, and 5 offered tests other than those recommended by Australian testing guidelines (eg, *Ureaplasma*). Nine services (1 SNP, 8 self-test) had no minimum age requirements for access. Reliability scores (scale 0-8) were similar between all services (range 4.75-8.0). Quality weighted scores (scale 0-58) were similar between SNP and SS services (average 44.89, SD 5.56 and 44.75, SD 1.77, respectively) but lower for ST services (22.66, SD 8.93; *P*=.002). Government-funded services were of higher quality than private services (43.54, SD 6.71 vs 29.43, SD 13.55; *P*=.03). The cost for services varied between SNP (Aus $0-$595; ie, US $0-$381.96), self-sample (Aus $0; ie, US $0), and ST (Aus $0-$135; ie, US $0-$86.66). The time to test results was much shorter for SNP services (~4 days) than for SS (~12 days) and ST (~14 days).

**Conclusions:**

This review identified considerable variability in the quality and reliability of the web-based STI/HIV testing services in Australia. Given the proliferation and use of these services will likely increase, it is imperative that Australia develops national standards to ensure the standard-of-care offered by web-based STI/HIV testing services is appropriate to protect Australian users from the impact of poorly performing and inappropriate tests.

## Introduction

Sexually transmitted infection (STI) rates continue to increase across Australia with gonorrhea up 150%, chlamydia up 17%, and syphilis up more than 250% in the 10 years prior to the COVID-19 pandemic commencing in 2020 [1]. Left untreated, STIs can have considerable morbidity including pelvic inflammatory disease, infertility, and neurological disease, and if transmitted from mother to baby, can cause miscarriage and stillbirth [2]. Timely access to testing and treatment is crucial to reduce STI/HIV transmission but can be hindered by factors like service availability stigma and lack of knowledge [3]. In response to growing demand and the impact of the COVID-19 pandemic restricting access to in-person services [4], web-based STI/HIV testing services have proliferated in recent years particularly in the United Kingdom [5] and Canada [6]. Web-based services can include internet-only services that have no direct communication with users, telehealth services, or a combination of both.

Although web-based STI/HIV services provide benefits such as privacy and convenience [7], they have drawbacks such as not being able to see a provider in person, trust, and challenges for those with low health or digital literacy [7,8]. Most in-person primary care services in Australia and telehealth consultations supported by these services undergo accreditation to meet minimum safety and quality standards [9]. However, other than the Australian Health Practitioner Regulatory Agency and National Board’s guidance for Telehealth services and Medical Board of Australia guidance for technology-based patient consultations dating back to 2012, there is little guidance available about what makes a quality web-based STI/HIV service in Australia where the service is provided wholly on the internet [10,11]. Without proper guidance inappropriate testing and treatment, inadequate follow-up and no statutory reporting of infections can occur [12]. In response to these concerns, the United Kingdom has introduced standards for web-based STI/HIV testing services that are (1) safe; (2) effective; (3) treat people with kindness, respect, and compassion; (4) responsive; and (5) have adequate governance and leadership [13].

Given the growing availability of web-based STI/HIV testing services and the lack of Australian guidance for such services, this study aimed to identify and assess the quality of available services in Australia. We developed a quality assessment checklist informed by the UK standards [13], reviews of web-based STI/HIV services available overseas [5,14], and adherence to the Australian STI Management Guidelines [15] and the Therapeutic Goods Administration (TGA) [16]. We also assessed services for their transparency in reliability and credibility using the Health on the Net Foundation code of conduct (HONcode) [17].

## Methods

### Systematic Search

We undertook a systematic Google search from March 3 to 5, 2022, using Boolean operators and terms related to test service (online/home/instant/rapid), STIs (sti/std/chlamydia/gonorrhoea/HIV/syphilis/herpes), and test type (test/self-test/diagnostic/kit). This resulted in 6 separate searches with terms within a group separated by “or” and terms between groups separated by “and,” as shown in Multimedia Appendix 1. As the market is rapidly evolving, we repeated the search on September 14, 2022, to identify any new services.

### Inclusion Criteria

While most Google users only look at the first page of search results [18], we screened the first 10 web pages (~100 results) from each search by title and description, and services were eligible for inclusion if they provided a web-based STI/HIV testing service within Australia (must test for any chlamydia, gonorrhea, syphilis, or HIV). While web-based services can integrate telehealth, this study excluded services that required telehealth to order STI/HIV tests. Including all telehealth services would be too broad and could apply to the majority of general practices across Australia where telehealth services are offered.

### Categorization

Upon selection, we classified web-based testing services as (1) self-testing (ST) where an individual could order and receive a test kit at home, self-collect specimens, test, and interpret the results themselves [19]; (2) self-sampling (SS) where an individual orders a collection kit to collect specimens at home and return specimens to the laboratory via mail, drop off, or courier for testing and interpretation [19]; or (3) self-navigated pathology (SNP) where the web-based service generates the appropriate documentation (eg, pathology test request) to provide to a pathology collection center for specimen collection, testing, and interpretation. For each service we identified whether the service (1) was funded by the government and free to users, or provided by a private company at a cost to the user, (2) charged a fee for their services to the user, (3) billed services to Medicare (Australia’s universal health insurance scheme), (4) provided information on the website in languages other than English, and (5) had a minimum age requirement to use the service. This latter criterion is important because of Australia’s laws around the age of consent and mandatory reporting of underage sexual activity.

### Reliability Assessment

Consistent with reviews of overseas web-based STI/HIV testing services [20], we calculated a reliability and credibility score using the HONcode. The HONcode “promotes the effective and reliable use of the new technologies for telemedicine in healthcare around the world” [17]. It is the most widely used tool for determining the reliability of health information on the internet [21] by assessing against 8 principles (ie, authority, complementarity, privacy, attribution, justification, contact details, financial disclosure, and advertising policy). Each site was given a score for each principle (1=met principle; 0=not met) with a maximum score of 8. A summary of results and a brief description of these principles are in Multimedia Appendix 2.

### Quality Assessment

We developed a quality checklist for the information and services provided by each website using testing recommendations from the Australian STI Management Guidelines [15], whether the tests offered were approved by the TGA [16], quality indicators from current reviews of web-based STI/HIV testing services available overseas [5,14], and quality service recommendations from the UK standards [13]. The domains in the checklist included pretesting, usability, testing, and follow-up. Within each domain, we identified a number of criteria against which to assess the performance of the service. For example, under the pretesting domain, we assessed whether the service provided accurate health promotion information about STI/HIV and collected sexual history information to guide appropriate testing. Under the testing domain, we assessed whether the testing prescribed was consistent with guideline recommendations including testing for chlamydia, gonorrhea, syphilis, and HIV, and whether they prescribed testing of genital, anorectal, and oropharyngeal sites, depending on users’ sexual practices. The services were assessed against all domains scoring between 0 and 1 for each criterion (1=met all criteria with incremental values [0.25, 0.5, and 0.75] depending on the criteria). In total, 6 clinicians from the Australasian sexual health clinical leaders’ group, a national group of sexual health clinicians, assessed the checklist and identified which criteria were most important relative to another, to produce an overall weighted quality score with a maximum score of 58. The quality checklist with questions, description, and weights can be found in Multimedia Appendix 3.

We calculated the mean and median HONcode and quality scores for each service and compared the scores between services providing ST, SS, or SNP and between government or privately funded services using the appropriate nonparametric test (Kruskal Wallis or Mann Whitney *U* test). Analyses were conducted using Stata 17 (StataCorp).

### Cost

We also assessed each service for accessibility—namely, their costs and timely access, particularly rural access. Cost, in Australian dollars, was the total out-of-pocket cost for the service. Where possible, members of the research team requested or purchased STI/HIV tests or self-test kits from the web-based services. All monetary values are represented in Australian dollars, which had a conversion rate of 0.64 to the United States dollar at the time of this study.

### Timely Access

We calculated the total time it took from requesting or purchasing the test on the internet to obtaining the test result, including shipping and laboratory processing time. As the research team was based in Melbourne, Victoria, our estimates of time were limited to accessing testing available in Melbourne and only for ST and SNP services. We did not test SS services as these are only available in a few Australian regions outside Melbourne.

### Rural Access

Rural availability was determined only for SNP as postal services are available throughout Australia providing ready access to self-tests and SS and was defined as the travel time (via car or public transport in Google Maps) for a person to access a pathology collection center if they were based in a particular postcode. To determine the postcodes for this analysis, we used the Australian Bureau of Statistics remoteness area data to classify postcodes as remote or very remote. We then calculated the median population across all postcodes within both remoteness areas and identified the postcode in which the population was closest to the overall median population. This postcode was selected for inclusion in this analysis. We selected 1 postcode within each state or territory, excluding the Australian Capital Territory which has no rural postcodes.

### Ethical Considerations

As this was a review of publicly available websites of STI/HIV testing services, there are no ethical concerns and human ethics approval was not required.

## Results

### Systematic Search and Categorization

In total, 6 Boolean searches conducted during March 2022 identified 13 web-based STI/HIV services in Australia with another 4 identified in September 2022. The 17 services were categorized as ST (2 government, 6 private), SS (2 government), and SNP (2 government, 5 private; Table 1). Among the 6 government funded, only 1 offered testing for all 4 infections (chlamydia, gonorrhea, syphilis, and HIV) including genital, anorectal, and oropharyngeal sites, with 3 services offering chlamydia and gonorrhea testing only and 2 services offering HIV testing only. Among the 11 privately funded services, only 3 services offered testing for all 4 infections including genital, anorectal, and oropharyngeal sites. All 9 SS and SNP services provided options for telehealth consultation to discuss results. Overall, 7 services (2 SS and 5 SNP) required a minimum age of 16 or 18 years to access testing, 1 SNP service had no age restrictions, and 1 was only available to existing clients of its in-person service. None of the ST services had a minimum age for purchasing tests. Only 1 service provided information in a language other than English.

**Table 1:** Summary of available web-based services for STI^a^/HIV testing in Australia in 2022 including funding type, test costs, infection sites tested, and Medicare requirements.

Provider (website name)	Funding type	Category	Routine STI testing cost (Aus $^b^)						Medicare^c^
			HIV	Syphilis	Chlamydia	Gonorrhea	All 4		
InstantScripts	Private	SNP^d^	20	20	20 (A^e^, O^f^, FU^g^, HVS^h^)	20 (A, O, FU, HVS)	20	Required	
StigmaHealth	Private	SNP	38.99 (A, O, FU, HVS)	38.99 (A, O, FU, HVS)	38.99 (A, O, FU, HVS)	38.99 (A, O, FU, HVS)	38.99	Required	
SmartHealth	Private	SNP	55	55	65 (FU)	65 (FU)	95	No	
MyCheck, Sydney Sexual Health Centre	Government	SNP	0 (A, O, FU, HVS)	0 (A, O, FU, HVS)	0 (A, O, FU, HVS)	0 (A, O, FU, HVS)	0	No	
WA Health (Could I have it?, Get the Facts, Health Sexual)	Government	SNP	—^i^	—	0 (FU)	0 (FU)	—	No	
iMedical	Private	SNP	25	22	72 (FU and O)	72 (FU and O)	119	No	
Better2Know	Private	SNP	195	395 (A, O, FU, HVS)	395 (A, O, FU, HVS)	395 (A, O, FU, HVS)	595	No	
13 HEALTH Webtest	Government	SS^j^	—	—	0 (FU)	0 (FU)	—	No	
TESTme	Government	SS	—	—	0 (FU)	0 (FU)	—	No	
Buy STD Test Kits	Private	ST^k^	49.95	84.95(US^l^ and HVS)	84.95(US^l^ and HVS)	84.95(US^l^ and HVS)	134	No	
Test Kit Labs	Private	ST	—	74.95(US and HVS)	74.95(US and HVS)	74.95(US and HVS)	—	No	
LT Labs	Private	ST	29	29	29 (US and HVS)	29 (US and HVS)	99	No	
HIV Test Australia	Private	ST	43.95	28.95	28.95 (US and HVS)	28.95 (US and HVS)	124.95	No	
Test Kit Mart	Private	ST	44	44	44 (US and HVS)	44 (US and HVS)	114	No	
Atomo Diagnostics	Private	ST	39	—	—	—	—	No	
SA MESH	Government	ST	0	—	—	—	—	No	
Rapid (Brisbane)	Government	ST	0	—	—	—	—	No	

^a^STI: sexually transmitted infection.

^b^Aus $1=US $0.64.

^c^Those labeled “required” mean it is necessary to be Medicare eligible (Australia’s universal health insurance scheme available to all Australian residents) to use the service. “No” means Medicare eligibility is not required to use this service.

^d^SNP: self-navigated pathology.

^e^A: anorectal swab.

^f^O: oral swab.

^g^FU: first urine.

^h^HVS: high vaginal swab.

^i^“—”: Aus $ amount is not unknown or unavailable.

^j^SS: self-sampling.

^k^ST: self-testing.

^l^US: urethral swab.

### Reliability Assessment

HONcode scores ranged from 4 to 8 with only 1 website meeting all principles for reliable, credible health information. Overall scores among the 3 categories were similar: SNP (mean 5.29, SD 0.78; median 5, IQR 5-6), self-sample (mean 6.00, SD 0; median 6, IQR 6-6), and self-test (mean 4.75, SD 1.39; median 4, IQR 4-5; *P*=.12). Scores were higher for government-funded (mean 5.67, SD 0.52; median 6, IQR 5-6) compared with private services (mean 4.82, SD 1.25; median 4, IQR 4-5; *P*=.03; Table 2).

**Table 2:** Summary of the assessment of the quality and reliability of each web-based STI^a^/HIV testing service available in Australia in 2022.

Provider (website name)		Category	Quality score (range 0-58)	Reliability score^b^ (range 0-8)
InstantScripts		SNP^c^	46.25	5
StigmaHealth		SNP	48.25	5
SmartHealth		SNP	39.50	6
MyCheck, Sydney Sexual Health Centre		SNP	54.25	6
WA Health (Could I have it?, Get the Facts, Health Sexual)		SNP	44.75	6
Better2Know		SNP	43.75	5
iMedical		SNP	37.50	4
13 HEALTH Webtest (QLD health)		SS^d^	46.00	6
TESTme		SS	43.50	6
Buy STD Test Kits		ST^e^	18.75	4
HIV Test Australia		ST	16.25	4
Test Kit Labs		ST	14.00	4
Test Kit Mart		ST	17.75	4
LT Labs		ST	17.75	4
Atomo Diagnostics		ST	24.00	8
SA MESH		ST	36.75	5
Rapid (Brisbane)		ST	36.00	5
**Self-navigated pathology group**		SNP		
	Mean (SD)		44.89 (5.56)	5.29 (0.78)
	Median (IQR)		44.75 (39.50-48.25)	5 (5-6)
**Self-sample group**		SS		
	Mean (SD)		44.75 (1.77)	6.00 (0)
	Median (IQR)		44.75 (43.50-46.00)	6 (6-6)
**Self-test group**		ST		
	Mean (SD)		22.66 (8.93)	4.75 (1.39)
	Median (IQR)		18.25 (17.00-30.00)	4 (4-5)
**Government**		N/A^f^		
	Mean (SD)		43.54 (6.71)	5.67 (0.52)
	Median (IQR)		44.13 (36.75-46.00)	6 (5-6)
**Private**		N/A		
	Mean (SD)		29.43 (13.55)	4.82 (1.25)
	Median (IQR)		24.00 (17.75-43.75)	4 (4-5)

^a^STI: sexually transmitted infection.

^b^HONcode: Health on the Net Foundation code of conduct.

^c^SNP: self-navigated pathology.

^d^SS: self-sampling.

^e^ST: self-testing.

^f^N/A: not applicable.

### Quality Assessment

The quality score for each service ranged from 14.00 to 54.25, with none satisfying all elements of a quality service. SNP (mean 44.89, SD 5.56; median 44.75, IQR 39.50-48.25) and SS services (mean 44.75, SD 1.77; median 44.75, IQR 43.50-46.00) scored similarly while ST services scored much lower (mean 22.66, SD 8.93; median 18.25, IQR 17.00-30.00; *P*=.002). Among the 7 SNP pathologies, 6 had lower quality scores for not obtaining a full patient history, 5 offered tests not recommended by STI guidelines (eg, *Ureaplasma* or herpes), and 1 service provided an e-Script for chlamydia treatment only. Both SS services had lower quality scores for not offering all recommended tests and not testing all appropriate infection sites, as they only offered urine testing for chlamydia and gonorrhea. ST services had lower quality scores for failing to meet the criterion across all domains. Two-thirds (6 of 9) of these services provided ST devices not registered by TGA for use in Australia. The only TGA-registered home test for STI/HIV in Australia is the HIV self-test manufactured by Atomo Diagnostics. Government-funded services (mean 43.54, SD 6.71; median 44.13, IQR 36.75-46.00) scored higher overall compared with private (mean 29.43, SD 13.55; median 24.00, IQR 17.75-43.75; *P*=.003; Table 2; Multimedia Appendix 4).

### Cost

Service costs in Australian dollars varied among the 3 categories: SNP (Aus $0-$595; ie, US $0-$381.96), self-sample (Aus $0; ie, US $0), and self-test (Aus $0-$135; ie, US $0-$86.66). The cost was very dependent on the service’s source of funding with those government-funded having no cost while private services cost between Aus $20 (US $12.83) and Aus $595 (US $381.96; Table 1). For 2 of the privately funded SNP services, users paid a fee to use the service (Aus $20-$38.99; ie, US $12.83-$25.01) and were also required to have a valid Medicare card with tests billed to Medicare.

### Timely Access

SNP took the shortest time to obtain a test result at about 4 days. SS took an estimated 12 days. ST took the longest at an estimated 14 days.

### Rural Access

Travel times to access STI/HIV testing ranged from 1 to 650 minutes by car and 1 minute to no access by public transport and varied considerably between states and the Northern Territory (Table 3).

**Table 3:** Summary of rural access for self-navigated pathology STI^a^/HIV testing services available in Australia in 2022 using 1 postcode to represent populations in “remote” and “very remote” areas (according to the Australian Bureau of Statistics) in each state or territory. Australian Capital Territory is excluded because it does not include any remote or very remote postcodes.

Provider	Rural access by state (minutes)																			
	Victoria			New South Wales			Queensland			South Australia			Western Australia			Tasmania			Northern Territory	
	Car^b^	PT^c^	Car		PT	Car		PT	Car		PT	Car		PT	Car		PT	Car		PT
InstantScripts	15	28	1		6	199		/^d^	454		/	205		/	2		6	3		/
StigmaHealth	15	28	1		6	199		/	454		/	205		/	2		6	3		/
SmartHealth	77	104	131		170	204		/	/		/	281		/	2		6	650		/
MyCheck	—^e^	—	131		170	—		—	—		—	—		—	—		—	—		—
WA Health	—	—	—		—	—		—	—		—	258		/	—		—	—		—
Better2Know	/	104	/		/	/		/	/		/	/		/	2		6	/		/
iMedical	15	28	1		6	199		/	454		/	205		/	2		6	3		/

^a^STI: sexually transmitted infection.

^b^Car: time taken to travel by car for STI/HIV testing.

^c^PT: time taken to travel by public transport for STI/HIV testing.

^d^“/” is used to indicate “access not possible.”

^e^“—” is used to indicate when the service is not available in the state or territory.

## Discussion

### Principal Results

We identified 17 services that provided web-based STI/HIV testing in Australia. We found considerable variability in quality and adherence to the reliability and credibility standards of the HONcode. SNP provided the fastest testing service but often at a substantial cost or requiring the user to have a Medicare card. Of the 2 free SNP services, only 1 provided testing for the 4 STI/HIV at all infection sites, and both were only available for those living in Western Australia and New South Wales. While the 2 SS services were free, they only tested for chlamydia and gonorrhea and were only available in limited areas in Victoria and Queensland. Of concern, several services neither provided health promotion material nor collected sufficient clinical information to test for the appropriate infections at the appropriate infection sites. Furthermore, some services tested for infections, not in the STI guidelines (eg, *Ureaplasma*), and several self-tests were not approved for use in Australia. Users of these services are at risk of having inadequate and inappropriate testing, often with poor test performance, producing incorrect results, and receiving insufficient health information.

### Comparison With Prior Work

Previous studies have shown that access to services is a significant barrier to testing [22,23], and we found that even with the provision of web-based services, people living in some rural areas must travel great distances to find a pathology collection center. While postal specimen collection kits may be a solution for users in rural areas, return rates of less than 60% reported in previous Australian studies [24], can make these services unsustainable. However, studies of web-based testing services in London [25] and Amsterdam [26] report kit return rates of 80%, offering optimism for the future of web-based services in Australia, particularly as our existing specialist STI services are at capacity [27].

### Implications of Results

Our study found that Medicare-ineligible individuals (eg, international students and temporary visa holders) can be disadvantaged when accessing web-based STI/HIV testing services in Australia because they can be excluded for several reasons including lack of Medicare card, poor English literacy, or they need to pay potentially substantial amounts of money, depending on the service accessed. However, this also applies to their access to in-person services in Australia. This is concerning as previous studies have reported population groups such as Medicare-ineligible men who have sex with men, often have higher rates of STI/HIV making it important for them to have ready access to affordable STI/HIV testing, including via web-based services like those assessed in this study [28]. We also found that several services did not provide STI testing at the anorectal or oropharyngeal sites as is recommended by Australian STI guidelines for men who have sex with men [15], further disadvantaging users. Only 1 service provided information in languages other than English potentially excluding those from non–English-speaking backgrounds.

While in-person sexual health services in Australia are accredited to meet minimum safety and quality standards [9], the Australian Health Practitioner Regulation Agency only regulates individual practitioners and not websites, and the TGA only regulates services and goods provided on Australian-based domains. Our review highlights the urgent need for standards for web-based STI/HIV testing services accessible in Australia, including those based overseas. The Faculty of Sexual and Reproductive Healthcare and the British Association for Sexual Health and HIV in the United Kingdom [13] have produced standards for the web-based provision of sexual and reproductive health services to be used by providers of web-based services and to enable users to understand what to expect from the web-based provider. Our quality checklist captured the UK standards but was more comprehensive in line with similar reviews overseas [5,14]. The UK standards place considerable emphasis on patient safety including identifying and managing vulnerable populations (eg, children), requiring them to have a telehealth consultation. We were very concerned to identify several services available for those the age of 16 years or younger, placing these users at risk of harm in the absence of at least a telehealth consultation.

### Limitations

This study had several limitations: (1) we limited our review to the first ten internet pages per search and it is possible that some services were missed considering the use of paid advertisements has the potential to skew results toward private services; (2) while we requested or purchased STI/HIV tests from the services to help us assess service quality and accessibility, we were unable to do this for all services because of geographical restrictions; (3) our assessment of rural accessibility was limited to one postcode per state or territory to simplify our calculations and this will both under- and overestimate the time taken to travel for STI/HIV testing. Nevertheless, it does highlight that there are rural accessibility issues throughout Australian states and territories; and (4) our review did not capture time to treatment and prompt treatment is vital to reduce ongoing transmission. It is likely that any positive test results obtained from the ST services identified in our review would need to be laboratory-confirmed before treatment could be prescribed, adding further delays to treatment for users of these services.

### Conclusions

Access to web-based STI/HIV services in Australia is growing, and the proliferation and use of these services will likely increase given our specialist STI services are at capacity [27,29]. Given the variability in quality and reliability of available services, it is imperative that Australia develops national standards for providers and information for users to ensure that the standard of care is appropriate to protect Australian users from the repercussions of poorly performing and inappropriate tests and suboptimal management. Further, the role of web-based services in the broader health care environment needs to be considered more widely, particularly in the context of quality, governance and government regulation, equity, and funding mechanisms.

## Appendix

Multimedia Appendix 1 Search combinations using Boolean operators to separate terms related to test service –(online/home/instant/rapid); STIs –(sti/std/chlamydia/gonorrhoea/HIV/syphilis/herpes); and test type –(test/self-test/diagnostic/kit) and the number of services identified from each search using Google.

Multimedia Appendix 2 Summary of reliability scores using the HONcode for each available web-based service for STI/HIV testing in Australia in 2022.

Multimedia Appendix 3 Sexual health clinician weighted scorecard developed based on the Australian STI Management Guidelines, TGA Guidelines, reviews of web-based STI/HIV testing services available overseas and UK standards to assess quality of available web-based services for HIV/STI testing in Australia in 2022.

Multimedia Appendix 4 Detailed quality scores for each available web-based STI/HIV testing service in Australia in 2022.

## Abbreviations

HONcode: Health on the Net Foundation code of conduct
SNP: self-navigated pathology
SS: self-sampling
ST: self-testing
STI: sexually transmitted infection
TGA: Therapeutic Goods Administration

## Data Availability

All data generated or analyzed during this study are included in this published paper and its Multimedia Appendix information files.
