# Supplementary material for: Web-Based STI/HIV Testing Services Available for Access in Australia: Systematic Search and Analysis
Source: J Med Internet Res. 2023 Sep 22;25:e45695. doi: 10.2196/45695 (PMC10559186; doi:10.2196/45695)
Supplement: Multimedia Appendix 1 [file jmir_v25i1e45695_app1.pdf]

Multimedia Appendix 1: Search combinations using Boolean operators to separate terms related to test service –(online/home/instant/rapid); STIs –(sti/std/chlamydia/gonorrhoea/HIV/syphilis/herpes); and test type –(test/self-test/diagnostic/kit) and the number of services identified from each search using Google.

**Boolean search**	**Term 1 (AND)**	**Term 2 (AND)**	**Term 3**	**Service category**		
				Self-testing	Self-sampling	Self-navigated pathology
#1	"online" OR "home" OR "instant" OR "rapid"	"sti" OR "std"	"test"	5	0	6
#2	"online" OR "home" OR "instant" OR "rapid"	"chlamydia" OR "gonorrhea" OR "HIV" OR "syphilis" OR "herpes"	"test"	4	1	1
#3	"online" OR "home" OR "instant" OR "rapid"	"sti" OR "std"	"test" OR "self-test" OR "diagnostic"	4	1	4
#4	"online" OR "home" OR "instant" OR "rapid"	"chlamydia" OR "gonorrhea" OR "HIV" OR "syphilis" OR "herpes"	"test" OR "self-test" OR "diagnostic"	5	1	3
#5	"sti" OR "std"	"test" OR "self-test" OR "diagnostic"	"kit"	3	0	1
#6	"chlamydia" OR "gonorrhea" OR "HIV" OR "syphilis" OR "herpes"	"test" OR "self-test" OR "diagnostic"	"kit"	3	1	2
			**Total**	**8**	**2**	**7**
