# Supplementary material for: Web-Based STI/HIV Testing Services Available for Access in Australia: Systematic Search and Analysis
Source: J Med Internet Res. 2023 Sep 22;25:e45695. doi: 10.2196/45695 (PMC10559186; doi:10.2196/45695)
Supplement: Multimedia Appendix 2 [file jmir_v25i1e45695_app2.pdf]

Multimedia Appendix 2. Summary of *reliability* scores using the HONcode for each available web-based service for STI/HIV testing in Australia in 2022.

**Website Name**	**InstantScripts**	**StigmaHealth**	**SmartHealth**	**Sydney Sexual Health Center**	**WA Health (Could I have it?, Get the Facts, Health Sexual)**	**iMedical**	**Better2K0w**	**13 HEALTH Webtest (QLD health**	**TESTme**	**Buy STD Test Kits**	**Test Kit Labs**	**LTLabs**	**HIV Test Australia**	**Test Kit Mart**	**Atomo Diag0stics**	**SA MESH**	**Rapid (Brisbane)**
**Principle 1: Authority**<br>Give qualifications of authors	1	0	1	1	1	0	1	1	1	0	0	0	0	0	1	0	0
**Principle 2: Complementarity**<br>Information to support, not replace	1	1	1	1	1	1	1	1	1	1	1	1	1	1	1	1	1
**Principle 3: Confidentiality**<br>Respect the privacy of site users	1	1	1	0	1	1	1	1	1	1	1	1	1	1	1	1	1
**Principle 4: Attribution**<br>Cite the sources and dates of medical information	0	1	0	1	0	0	0	0	0	0	1	0	0	0	1	0	0
**Principle 5: Justifiability**<br>Justification of claims / balanced and objective claims	1	1	1	1	1	1	0	1	1	0	0	0	0	0	1	1	1
**Principle 6: Transparency**<br>Accessibility, provide valid contact details	1	1	1	1	1	1	1	1	1	1	1	1	1	1	1	1	1
**Principle 7: Financial disclosure**<br>Provide details of funding	0	0	1	1	1	0	1	1	1	1	0	1	0	1	1	1	1
**Principle 8: Advertising**<br>Clearly distinguish advertising from editorial content	0	0	0	0	0	0	0	0	0	0	0	0	1	0	1	0	0
**Total Score out of 8**	**5**	**5**	**6**	**6**	**6**	**4**	**5**	**6**	**6**	**4**	**4**	**4**	**4**	**4**	**8**	**5**	**5**
