# Supplementary material for: Web-Based STI/HIV Testing Services Available for Access in Australia: Systematic Search and Analysis
Source: J Med Internet Res. 2023 Sep 22;25:e45695. doi: 10.2196/45695 (PMC10559186; doi:10.2196/45695)
Supplement: Multimedia Appendix 3 [file jmir_v25i1e45695_app3.pdf]

Multimedia Appendix 3. Sexual health clinician weighted scorecard developed based on the Australian STI Management Guidelines, TGA Guidelines, reviews of web-based STI/HIV testing services available overseas and UK standards to assess *quality* of available web-based services for HIV/STI testing in Australia in 2022.

Provider					
**Indicator**	**Explanation**	**Answer**	**Rubric**	**Clinical Weight**	**Notes**
**Pre-test**					
Information	Does the website give accurate **health promotional** information about the STIs?	Yes	1	**3**	
		No	0		
Patient History	Did the provider collect a **patient history** pertinent to the STI test?	Adequate	1	**5**	This can include any question that assesses the individual's risk for infection. Websites were deemed 'Adequate' if they asked a question about men who have sex with men and a question about Aboriginal and Torres Strait Islander. 'Inadequate' means that the website asked questions, but not specifically about MSM and Aboriginality.
		Inadequate	0.5		
		No	0		
Testing at Appropriate Sites	Does the provider discuss **infection at different sites (oral, anal, vaginal, etc.)** and/or provide appropriate testing at these sites?	Both	1	**5**	Some providers may only provide information about infection/testing at a different site, but not offer specimen collection at these sites.
		Information only	0.5		
		Neither	0		
**Testing**					
Tests Available	Are all tests recommended for a **routine STI checkup** (chlamydia, gonorrhea, syphilis, and HIV) available on the web-based platform?	All	1	**5**	Some web-based services may only provide one or some of the recommended tests, while others will provide all 4.
		Some	0.5		
		Only one	0.25		
		None	0		
		Yes	1	**4**	

Unnecessary Testing	Does the provider only promote testing for STIs recommended by **Australian STI management guidelines**?	No	0		Some services may promote routine testing for STIs that are not currently recommended for routine testing (e.g. herpes, etc.)
Test TGA Approval	Is the test offered **Therapeutic Goods Administration (TGA)** approved?	Yes	1	**5**	We will assume that any service that requires the use of a laboratory to analyze a sample is TGA approved.
		No	0		
Test Type	Does the **test type** offered reflect those recommended by the Australian STI management guidelines (NAAT, etc.)?	Yes	1	**4**	We will assume that any service that requires the use of a laboratory to analyze a sample meets the guidelines.
		No	0		
**Usability**					
Instructions	Are their **instructions** provided for the test or process?	Yes	1	**4**	This would vary depending on test type;<br>Self-navigated pathology- the process for obtaining a pathology form to take to a pathology and then getting result<br>Self-sampling- the process of receiving the testing kit, instructions to self-collect samples, sending kit back to lab and then getting result<br>Self-testing- instructions on how to use point of care tests at home
		No	0		
Support Contact	Is there an option to **contact support** for further questions?	Yes	1	**3**	If yes, the contact options should be provided in the comments.
		No	0		
**Follow-up**					
Result Communication	How was the result provided?<br>Provider always initiated	Provider	1	**4**	Two methods (1) Provider initiated would involve active notification from a medical professional to the patient (phone call, SMS, email, etc.).<br>(2) Patient initiated would involve the patient actively
		Patient	0.5		

	OR<br>Patient initiated	No result	0		contacting the provider to receive the result (portal login, call, etc.) OR the patient interpreting their own test result.
Result Interpretation	Is the **result interpretation** easy?	Yes	1	**3**	The result was considered somewhat easy to interpret if it had to be self-interpreted by the individual.
		Somewhat	0.5		
		No	0		
Treatment	Is information and/or referral for **treatment** provided for a positive test?	Provided TX	1	**5**	There are different levels of what a website provides related to treatment with each building on the previous. (0.25) Websites only offered information on treatment; (0.5) provided a referral to see a GP to receive treatment; (0.75) provided a prescription for the patient to get treatment from a pharmacy; (1) filled the prescription and sent treatment to the person.
		Provided RX	0.75		
		Referral	0.5		
		Information	0.25		
		Nothing	0		
Partner Notification	Is there information on **partner notification** for positive tests? Are services provided?	Services	1	**4**	The website could offer their own notification service, provide information or links to other notification services, or offer nothing on partner services.
		Information, no service	0.5		
		None	0		
Disease Notification	Does the provider follow government **notifiable disease** guidelines and report positive results to the appropriate entity?	Yes	1	**4**	We will assume that any service that requires the use of a laboratory to analyze a sample, will also report diseases based on guidelines.
		No	0		
				**Total Quality Score**	**Max Score= 58**
