# Supplementary material for: Web-Based STI/HIV Testing Services Available for Access in Australia: Systematic Search and Analysis
Source: J Med Internet Res. 2023 Sep 22;25:e45695. doi: 10.2196/45695 (PMC10559186; doi:10.2196/45695)
Supplement: Multimedia Appendix 4 [file jmir_v25i1e45695_app4.pdf]

Multimedia Appendix 4: Detailed *quality* scores for each available web-based STI/HIV testing service in Australia in 2022.

**Indication**	**Explanation**	InstantScripts	StigmaHealth	SmartHealth	Sydney Sexual Health Center	WA Health	iMedical	Better2K0w	13 HEALTH Webtest (QLD health)	TESTme**or**	Buy STD Test Kits	Test Kit Labs	LTLabs	HIV Test Australia	Test Kit Mart	Atomo Diag0stics	SA MESH	Rapid (Brisbane)
Information	Does the website give accurate **health promotional** information about the STIs?	**3**	**3**	**3**	**3**	**3**	**3**	**3**	**3**	**3**	**3**	**3**	**3**	**3**	**3**	**0**	**3**	**3**
Patient History	Did the provider collect a **patient history** pertinent to the STI test?	**2.5**	**2.5**	**0**	**5**	**2.5**	**0**	**2.5**	**2.5**	**0**	**0**	**0**	**0**	**0**	**0**	**0**	**2.5**	**5**
Testing at Appropriate Sites	Does the provider discuss **infection at different sites (oral, anal,**	**5**	**5**	**0**	**5**	**2.5**	**2.5**	**5**	**2.5**	**0**	**2.5**	**0**	**0**	**0**	**0**	**0**	**2.5**	**0**

	**vaginal, etc.)** and/or provide appropriate testing at these sites?																	
Tests Available	Are all tests recommended for a **routine STI checkup** (chlamydia, gonorrhea, syphilis, and HIV) available on the web-based platform?	**5**	**5**	**5**	**5**	**2.5**	**5**	**5**	**2.5**	**2.5**	**5**	**2.5**	**5**	**5**	**5**	**1.25**	**1.25**	**1.25**
Unnecessary Testing	Does the provider only promote testing for STIs recommended by **Australian STI management guidelines**?	**0**	**0**	**0**	**4**	**4**	**0**	**0**	**4**	**4**	**0**	**0**	**0**	**0**	**0**	**4**	**4**	**4**
Test TGA Approval	Is the test offered **Therapeutic Goods Administration (TGA)** approved?	**5**	**5**	**5**	**5**	**5**	**5**	**5**	**5**	**5**	**0**	**0**	**0**	**0**	**0**	**5**	**5**	**5**
Test Type	Does the **test type** offered	**4**	**4**	**4**	**4**	**4**	**4**	**4**	**4**	**4**	**0**	**0**	**0**	**0**	**0**	**4**	**4**	**4**

	reflect those recommended by the Australian STI management guidelines (NAAT, etc.)?																	
Instructions	Are their **instructions** provided for the test or process?	**4**	**4**	**4**	**4**	**4**	**4**	**4**	**4**	**4**	**4**	**4**	**4**	**4**	**4**	**4**	**4**	**4**
Support Contact	Is there an option to **contact support** for further questions?	**3**	**3**	**3**	**3**	**3**	**3**	**3**	**3**	**3**	**3**	**3**	**3**	**3**	**3**	**3**	**3**	**3**
Result Communication	How was the result provided? Provider always initiated or patient initiated?	**4**	**4**	**4**	**4**	**4**	**4**	**4**	**4**	**4**	**0**	**0**	**0**	**0**	**0**	**0**	**0**	**0**
Result Interpretation	Is the **result interpretation** easy?	**3**	**3**	**3**	**3**	**3**	**3**	**3**	**3**	**3**	**0**	**1.5**	**1.5**	**0**	**1.5**	**1.5**	**3**	**1.5**
Treatment	Is information and/or referral for **treatment** provided for a positive test?	**3.75**	**3.75**	**2.5**	**1.25**	**1.25**	**0**	**1.25**	**2.5**	**5**	**1.25**	**0**	**1.25**	**1.25**	**1.25**	**1.25**	**2.5**	**1.25**

Partner Notification	Is there information on **partner notification** for positive tests? Are services provided?	**0**	**2**	**2**	**4**	**2**	**0**	**0**	**2**	**2**	**0**	**0**	**0**	**0**	**0**	**0**	**2**	**0**
Disease Notification	Does the provider follow government **notifiable disease** guidelines and report positive results to the appropriate entity?	**4**	**4**	**4**	**4**	**4**	**4**	**4**	**4**	**4**	**0**	**0**	**0**	**0**	**0**	**0**	**0**	**4**
**Total Quality Score (58)**		**46.25**	**48.25**	**39.5**	**54.25**	**44.75**	**37.5**	**43.75**	**46**	**43.5**	**18.75**	**14**	**17.75**	**16.25**	**17.25**	**24**	**36.75**	**36**
